# The mediating role of maladaptive cognitive schemas regarding the relationship between parenting styles and chronic pain in adolescents: a structural equation modelling approach

**DOI:** 10.1186/s13034-022-00496-5

**Published:** 2022-07-25

**Authors:** Saghar Salari, Maryam Shaygan, Giti Setoodeh

**Affiliations:** 1 Student Research Committee, Department of Psychiatric Nursing, School of Nursing and Midwifery, Shiraz University of Medical Sciences, Shiraz, Iran; 2 Community Based Psychiatric Care Research Center, School of Nursing and Midwifery, Shiraz University of Medical Sciences, P.O. Box 713451359, Shiraz, Iran; 3 Department of Psychiatric Nursing, School of Nursing and Midwifery, Shiraz University of Medical Sciences, Shiraz, Iran

**Keywords:** Adolescents, Chronic pain, Maladaptive cognitive schemas, Parenting styles

## Abstract

**Background:**

Although there is a growing body of evidence linking parenting styles to health outcomes, little emphasis has been dedicated to how parenting styles affect chronic pain in adolescents. Given the high prevalence of chronic pain in adolescents and taking into consideration the complexity of chronic pain and the factors affecting it, further research is needed to better understand the processes through which parenting styles affect adolescents’ pain. The purpose of the present study was to explore the mediating role of maladaptive schemas in the association between different parenting styles and chronic pain.

**Method:**

1302 adolescents aged 12 to 21 in Shiraz, Iran, were randomly selected to participate in this study. To identify adolescents with chronic pain, screening questions based on the 11th revision of the International Classification of Diseases were used. Buri’s Parental Authority Questionnaire (PAQ), and Young’s Schema Questionnaire-Short Form (YSQ-SF) were used to assess the parenting styles and maladaptive cognitive schemas, respectively. The structural equation modeling approach was carried out to evaluate the direct, indirect, and total effects of different parenting styles on chronic pain.

**Results:**

The results in the SEM models revealed that disconnection/ rejection (β = − 0.043, 95%CI =  − 0.07 to  − 0.02), impaired autonomy/ performance (β =  − 0.01, 95%CI =  − 0.02 to -0.003), over-vigilance/inhibition (β =  − 0.007, 95%CI =  − 0.01 to  − 0.008), and impaired limits schemas (β =  − 0.004, 95%CI =  − 0.006 to  − 0.002) significantly mediated the protective effects of the authoritative parenting style on chronic pain. It was also found that the mediating effects of disconnection/ rejection (β = 0.01, 95%CI = 0.01 to 0.02), and over-vigilance/ inhibition (β = 0.002, 95%CI = 0.001 to 0.02) existed in the relationship between the authoritarian style and chronic pain. The permissive style may also affect chronic pain through disconnection/ rejection (β = 0.004, 95%CI = 0.001 to 0.01), other-directedness (β = 0.01, 95%CI = 0.005 to 0.015), and impaired limits schemas (β = 0.05, 95%CI = 0.04 to 0.06).

**Discussion:**

The findings of the present study showed that maladaptive cognitive schemas play a mediating role in the relationship between parenting styles and chronic pain in adolescents. It seems that the interventions that target the effective communication between the parents and the adolescents can be considered as an important part in the chronic pain management in adolescents.

## Background

Chronic pain is defined as a pain that persists or recurs for more than 3 months, causing distress that interferes with performance [1]. Recent research shows a high prevalence of chronic pain among children and adolescents. A study conducted by Gobina et al., in 42 countries indicated that 44.2% of adolescents have suffered from a chronic pain on a weekly basis for the past 6 months [2]. Chronic pain in adolescents has some negative consequences such as depression, anxiety, decreased social activity, student absenteeism, decreased academic performance and reduced quality of life [3–5].

According to the bio-psycho-social model, the concept of pain is not just regarded as a sensory perception, but it is also considered as a complex situation that is affected by a wide range of psychosocial factors [6]. Among these factors, reference can be made to maladaptive behaviors in the family system [7]. In particular, some characteristics of parents such as parental distress or their emotional neglect are associated with chronic pain in adolescents [8, 9]. In one of their most recent studies, Shaygan and Karami indicated that some parenting styles, such as the authoritarian style could be considered as the predicting factors of chronic pain in adolescents [10]. However, few studies have yet examined the relationship between parenting styles and chronic pain in adolescents.

Parenting style is defined as a set of parental behaviors that influences the development of their children [11]. There are two main dimensions underlying parental behaviors; they are responsiveness and demandingness [12]. Responsiveness refers to the degree to which parents are accepting and sensitive to their children’s emotional and developmental needs [13, 14]. By contrast, demandingness refers to the extent to which parents control their child’s behavior or demand their maturity [13, 15]. In the 1960s, Baumrind [16] categorized three types of parenting styles based on the combination of the levels of responsiveness and demandingness: authoritative (balance between high levels of demandingness and high levels of responsiveness), authoritarian (highly demanding and unresponsive) and permissive (low levels of demandingness and high levels of responsiveness) [17]. Maccoby & Martin later added a fourth parenting style called Neglectful [11]. However, the three parenting styles approach has been extensively used until today, because the number of published instruments to measure parenting style is very few and most of them identify three styles instead of the four styles [18].

Culture plays a crucial role in the relationship between parenting styles and physical or psychological health in adolescents so that in some contexts specific parenting styles may be viewed as restrictive with potentially long-term damaging effects for children, but obsolete in others [19]. According to the findings of numerous studies, western societies have a tendency to favor the authoritative parenting style as the method that is most appropriate to raise healthy children [20–22]. In spite of the fact that Eastern and Western cultures are culturally distinct from one another, numerous studies have shown that some parenting styles undermine children’s physical and psychological development, both in Eastern and Western cultures [19, 20]. Furthermore, as the values and practices of the West spread across the globe, a growing number of young parents from a variety of cultures are also adopting such values in order to parent their children [20]. In fact, children and adolescents in different cultures require a balanced relationship with their parents where a balance between responsiveness and demandingness is managed (authoritative parenting) according to context and children and adolescent’s personal demands and needs [19, 20].

Many studies, including two recent studies in the Iranian context, have indicated that there is a positive relationship between the authoritative parenting style and children's physical health, while there is a negative relationship between the authoritarian and permissive parenting styles and children's physical health [10, 23–27]. Natalucci et al. in their narrative review, showed that some behaviors of parents, especially mothers, such as oppressive or overprotective behaviors have a strong relationship with the severity of headache attacks in their children [28]. In their recent study, Shibata and colleagues revealed that overprotection and low parental care during childhood is related to the development of chronic pain in adulthood [24]. These results may suggest that chronic pain may be more closely associated with these two parenting styles; the authoritative style reduces the probability of occurrence/severity of pain, while the authoritarian style increases the probability of occurrence and/or severity of pain. However, in a recent study, Patel indicated that parenting styles of the parents do not predict the children’s pain [26]. Another study, which compared the generic family functioning between five groups of children with five different chronic diseases and one group of healthy children revealed that families of children with and without chronic conditions do not differ significantly from each other regarding the generic family functioning [29]. In addition, it is not known exactly how and under what mechanisms the parenting styles of the parents can affect the chronic pain in adolescents [10]. Therefore, it seems necessary to conduct further studies on examining the mediating factors regarding the relationship between the parenting styles and chronic pain in adolescents.

Some theoretical models have tried to describe the role of parental behaviors and family factors in causing chronic pain in children [30]. Palermo and Chambers model claims that individual factors such as the adolescents' emotional functioning may play a mediating role regarding the relationship between family functioning and children's pain [31]. In supporting this model, Cunningham et al. showed that child pain catastrophizing mediates the relationship between the parental factors such as parental encouragement and monitoring or parental protection with functional abdominal pain-related disability levels [32]. Similarly, in our recent study on a different sample of adolescents, it has been shown that emotional functioning (including emotional intelligence and psychological distress) significantly mediated the effects of authoritative and authoritarian parenting styles on chronic pain [27].

One of the important factors that may affect emotional functioning in adolescents are maladaptive cognitive schemas [33]. Maladaptive schemas are stable relational patterns that develop through harmful childhood experiences with primary caregivers, especially with parents [34, 35]. Based on clinical experience, Young and colleagues conceptualized 18 different maladaptive schemas that are generally located in 5 domains of disconnection/ rejection, impaired autonomy/ performance, over vigilance/ inhibition, other-directedness and impaired limits [34]. Numerous studies have shown that authoritative parenting style predicts lower levels of maladaptive schemas and authoritarian parenting style predicts higher levels of maladaptive schemas [36–39].

On the other hand, some of the previous studies have shown a relationship between maladaptive schemas and pain. For example, the results of two recent studies revealed the mediating role of depression and mindfulness in the relationship between the maladaptive schemas and chronic pain [40, 41]. Also, Saariaho et al. in a study comprised of 271 first-visit chronic pain adult patients suggested that the co-occurrence of maladaptive schemas and depression seems to worsen the pain experience [42]. However, further studies with larger sample sizes and improved methodology using powerful statistical tools are necessary to provide a greater insight into influencing factors of chronic pain in adolescents.

## Current study

Although there is a growing body of evidence linking parenting styles to health outcomes, little emphasis has been dedicated to how parenting styles affect chronic pain in adolescents [43]. To our knowledge, only one original research has been published to investigate how parents raise their children affects chronic pain in adolescents [27].

A review study of the current state of research on pediatric pain has identified several limitations of the previous research in the field of pediatric pain, including the need for further research in between-person factors (such as family factors) related to the pediatric pain, consideration of relationships among various within- and between-person factors, and the utilization of more complex statistical modeling such as exploration of mediation and moderation [44]. Furthermore, previous literature has often focused on one medical chronic pain diagnosis at a time, which is useful in the understanding of that particular disorder but is difficult to generalize to chronic pain diagnoses in general. McKillop and Banez argue for a second generation of research in pediatric pain, particularly more sophisticated methodology that considers a combination of within- and between-person factors which would provide more information about the mechanisms of maintenance of chronic pain in children and adolescents [44].

Given the high prevalence of chronic pain in adolescents and taking into consideration the complexity of chronic pain and the factors affecting it, further research is needed to better understand the processes through which parenting styles affect adolescents’ pain in different. The purpose of the present study was to explore the mediating role of maladaptive schemas in the association between different parenting styles and chronic pain. We aim to investigate which parenting styles via which maladaptive schemas may influence chronic pain among adolescents. The current study inspected several correlations among studied variables using an advanced statistical tool (structural equation modeling). It provided a unique opportunity to test numerous hypotheses while controlling error at the same time. Based on previous research in the context of Iran, it was assumed that the maladaptive schemas would mediate the relationship between parenting styles and chronic pain in adolescents. Because Iran is one of the countries where recent research has shown the advantage of authoritative over authoritarian parenting style, the postulated relationships were assumed to be significant for authoritative and authoritarian parenting styles, as shown in our earlier studies using different samples [10, 27].

## Methodology

### Study design

The current study is a cross-sectional research using a structural equation modeling approach.

### Setting

The current study was conducted from January to June 2021 on adolescents aged 12 to 21 in Shiraz, the largest city in the southern part of Iran.

### Participants

1333 adolescents were randomly selected to participate in this study as the research sample population. Adolescents were included in the study if they met the following criteria: an age range of 12–21 years [45], studied at one of the schools or colleges in Shiraz, and the ability and willingness to complete an online questionnaire. Adolescents were excluded if they had been diagnosed with a physical illness not related to pain (such as multiple sclerosis, diabetes, cancer), had chronic mental illnesses or developmental disorders (according to the statement of the adolescent or his/ her parents), and had no parental contact.

### Sample size

Given that 20 people are needed to perform the structural equation modeling for each parameter [46, 47], and having at least 20 parameters in this study (chronic pain, cognitive schemas and parenting styles), the minimum sample population for adolescents with chronic pain was estimated to be 400 adolescents. Assuming that at least 30% of adolescents in Shiraz were suffering from a chronic pain [10], 1333 adolescents were selected using a multi-stage sampling method. In the end, 1302 people (Mean age: 16.87 ± 1.94) participated in the current study due to participant dropouts. Because of an insufficient power and sample size has consequences on statistical tests [48], power analysis for the correlation tests has conducted with G-Power analysis software (version 3.1.3) [49] in order to assure us that the size of the sample is adequate to detect a medium (0.30) effect size (q = 0.30) with a power of 0.95 (α = 0.05, 1 – β = 0.95) [27, 50].

### Sampling

Based on the number of high school students aged 12 to 18 vs. college students aged 18 to 21, 839 and 494 students, respectively, were randomly selected to participate in the current study (a total of 1333 adolescents).

For selecting 839 high school students, a multi-stage clustering sampling method was used. Thus, in order to do that, at first, among each of the 4 delivery areas in Shiraz, 4 schools (one all-female school and one all-male school from each of the first and second-rank high schools) were selected using a simple random sampling [16 schools in total]. Then, about 52 students were selected from each high school using the simple random sampling (selection of random numbers using a randomization software). To select 494 college students, at first, 17 faculties were selected in Shiraz, and then about 29 students were selected from each faculty using the same method as the one for high school students. It should be noted that the adolescents who did not meet the eligible criteria were replaced by other randomly selected adolescents.

### Data collection and ethical considerations

The current study started after receiving the ethical approval from the ethics committee of the Shiraz University of Medical Sciences (IR.SUMS.REC.1399.1033). First, all the randomly selected adolescents received a letter in person explaining the objectives of the research project, information confidentiality, anonymity, and voluntary withdrawal from the research project. Then, if each adolescent and his/her parent, in case of the adolescent being under 18 years old, has competently completed the consent form, the adolescent was then asked to complete the online questionnaires. If not completed, a reminder message was sent to the adolescent twice in order to minimize the possibility of participants’ dropout as much as possible. The researchers tried to increase the desire and cooperation of adolescents to participate in this study by explaining the objectives of the study and multiple follow-ups. All data were anonymously collected and encrypted.

### Instruments

In addition to the standard socio-demographic assessment (age, gender, birth order, parents’ job and educational level), the following variables were assessed:

### Chronic pain assessment

To identify adolescents with chronic pain, three screening questions were used based on the 11th revision of the International Classification of Diseases (ICD‐11) [1]:

1. Do you currently have pain, and if so is it permanent or intermittent?
2. Have you experienced this pain or discomfort for more than 3 months?
3. Does this pain and discomfort affect your life and activities?

The adolescents who answered yes to these three questions were asked to answer more questions about the underlying cause of the pain (including rheumatoid arthritis, injury, etc.), the frequency of the pain (response categories: permanent, one or more attacks per day, one or more attacks per week, one or more attacks per month) and pain history (the number of months they experienced the pain). The adolescents were also asked to report their average pain intensity over the past two weeks on a numerical rating scale from 0 (painless) to 10 (the worst imaginable pain) [51]. The adolescents completed the forms individually. In our previous study, the quantitative face validity of each item was confirmed by calculating the impact score of each item [10]. The impact scores showed that all the questions had a score equal to or greater than 1.5. Content validity was also evaluated both qualitatively and quantitatively. Content Validity Ratio (CVR) for the items was indicated to be a value between 0.87 and 1, which was higher than the minimum acceptable value. To test reliability using the retest method, 80 respondents answered the same questions for the second time two weeks later and all questions showed a correlation coefficient of 0.74 ≥ [10].

### Maladaptive cognitive schemas questionnaire

Maladaptive cognitive schemas in the adolescents were assessed using Young’s Schema Questionnaire-Short Form (YSQ-SF). This questionnaire includes 75 questions that are answered based on a 6-point Likert scale (1 = completely false about me to 6 = completely describes me). The YSQ-SF examines 15 initial maladaptive schemas in 5 domains, so that there are 5 questions for each schema. These 5 areas include: disconnection/ rejection (questions 1 to 25), autonomy/ impaired performance (questions 26 to 45), other-directedness (questions 46 to 55), over vigilance/ inhibition (questions 56 to 65), and impaired limits (questions 66 to 75). If the sum of the scores for each domain are greater than 25, it means that the maladaptive schema exists in that domain [52]. Numerous studies have shown that the YSQ-SF has good validity and reliability [53, 54].

In a study by Khosravani et al., the reliability (Cronbach’s alpha = 0.75–0.91), criterion, predictive, and discriminant validity of the Persian version of the instrument used in this study were confirmed [55]. This questionnaire indicated a good internal consistency in the present study (coefficient omega = 0.95 for the total questionnaire, 0.89 for disconnection/ rejection, 0.89 for impaired autonomy/ performance, 0.80 for other directedness, 0.81 for over vigilance/ inhibition, and 0.82 for impaired limits).

### Parenting styles questionnaire

Buri’s Parental Authority Questionnaire (PAQ) was used to assess the parenting styles [56]. This questionnaire was comprised of 30 items, 10 for each of permissive (e.g., “While I was growing up my mother felt that in a well-run home the children should have their way in the family as often as the parents do”), authoritative (e.g., “As I was growing up, once family policy had been established, my mother discussed the reasoning behind the policy with the children in the family”) and authoritarian (e.g., “Even if her children did not agree with her, my mother felt that it was for our own good if we were forced to conform to what she thought was right”) parenting styles. The pattern of answering the questions is based on a 5-point Likert scale (strongly disagree to strongly agree). It has to be noted that for the measure of parenting among adult children, we used the sentences in past tense. In the end, by summing the scores of the questions related to each parenting style, three separate scores were obtained. Determining the parenting style based on this scale depends on the highest score that the adolescent gains through responding to each subscale. Sub-scale scores range from 10 to 50. In a study by Buri, the reliability and validity of the instrument used in this study were confirmed. The PAQ appeared to have adequate internal consistency (Cronbach’s alpha = 0.74–0.87) and test–retest reliability ranged from 0.77–0.92 [56]. Several studies confirmed the validity and reliability (Cronbach’s alpha = 0.73–0.85) of the Persian version of the PAQ [57, 58]. This scale indicated adequate internal consistency (coefficient omega = 0.81 for the total questionnaire, 0.60 for the permissive style, 0.83 for the authoritarian style, and 0.78 for the authoritative style) in the present sample.

### Statistical analysis

Descriptive statistics were reported to describe the characteristics of the study population. Continuous variables were expressed as mean ± standard deviation and categorical variables as frequency (percentage).

Then the assumptions including normality distribution, missing data, outlier values and collinearity of variables were examined and compared. The normality of observations was investigated with histogram charts and Skewness and Kurtosis values [59]. Outliers were detected based on a constant score of 3.29. The percentage of missing data was 16% (n = 208), and they were replaced with the mean values [60]. Bivariate correlations using Pearson's correlation coefficient were calculated to assess the multicollinearity among variables [46]. SPSS version 22 (SPSS, Inc., Chicago IL, USA) was used to compute these analyses. A 5% type1 error was considered for the significance level.

The Structural Equation Modeling (SEM) was used to determine the direct, indirect, and total effects of independent variables (authoritative, authoritarian, and permissive parenting styles( on the dependent variable)chronic pain(. Three hypothesized path models were performed to evaluate each independent variable's effects on the chronic pain in the presence of five mediators: Disconnection/ rejection, impaired autonomy/ performance, other directedness, over vigilance/ inhibition, and impaired limits. Independent variables and mediators were continuous variables, and the chronic pain was a binary outcome variable, Yes/No.

We conducted the analyses with the weighted least squares (WLS) estimator because it does not assume normally distributed variables, thereby providing the best approach for categorical, binary or non-normal item responses, which is appropriate given our binary data on chronic pain [61]. For the examination of the indirect effects, we used a 5,000 bootstrap resamples in order to have nonbiased 95% confidence intervals [62]. The proposed approach by Hayes was applied to inference about the mediators. According to Hayes, if 0 is outside the 95% confidence interval, then the indirect effect is deemed to be statistically different from 0 with 95% confidence [62, 63]. In addition, to examine the model fit, several fit indices were considered: the chi-square test of model fit (nonsignificant chi-square value indicates good fit), Comparative Fit Index (CFI), Root Mean Square Error of Approximation (RMSEA), Tucker–Lewis Index (TLI), and Standardized Root Mean Square Residual (SRMR). The values of > 0.95 for CFI and TLI, < 0.05 for RMSEA and SRMR represent a good fit [64]. The software R version 4.0.5 with the lavaan package was utilized to run path models.

## Results

Of the 1333 invited adolescents, 31 (2.3%) did not return the self-report instruments or consent forms, leaving 1302 adolescents, with an overall response rate of 97.67%. Overall, a total of 1302 adolescents participated in this study. The mean age of the adolescents was 16.87 ± 1.94 years old, the majority of them were males (53.70%), and about 53.80% were firstborn. Most of the adolescents’ fathers (62.80%) and mothers (70.70%) had a diploma or lower education level. The highest percentage of fathers and mothers were the employed parents (80.40%) and housewives (80.10%), respectively (Table1).

**Table 1:** Demographic characteristics of adolescents (n = 1302)

Demographic variables		Value
Age	Mean ± SD	16.87 ± 1.94
Gender n (%)	Female	603 (46.30%)
	Male	699 (53.70%)
Birth order n (%)	1	700 (53.80%)
	2	365 (28.00%)
	3	166 (12.70%)
	≥ 4	71 (5.50%)
Father education n (%)	≤ diploma	818 (62.80%)
	Academic	484 (37.20%)
Mother education n (%)	≤ diploma	920 (70.70%)
	Academic	382 (29.30%)
Father job n (%)	Retired	193 (14.80%)
	Employed	1047 (80.40%)
	Unemployed	62 (4.80%)
Mother job n (%)	Housewife	1043 (80.10%)
	Employed	232 (17.80%)
	Retired	27 (2.10%)

The characteristics of pain in the adolescents are represented in Table 2. There were 219 (16.8%) adolescents who met the ICD-11 criteria of chronic pain. The mean value of pain intensity over the two weeks was 4.74 ± 2.48. Most of the adolescents with chronic pain experienced one or more pain attacks per month (35.2%). Headache (37.90%) and spinal column/ musculoskeletal pain (18.70%) were the most frequently reported pains.

**Table 2:** Pain characteristics in adolescents (n = 1302)

Pain characteristics	Values
Chronic pain	219 (16.80%)
Acute pain	33 (2.50%)
No pain	1050 (80.60%)
Pain intensity (Mean ± SD)	4.74 ± 2.48
Frequency of pain	
≥ 1 attack per day	32 (14.60%)
≥ 1 attack per week	66 (30.10%)
≥ 1 attack per month	77 (35.20%)
Permanent	14 (6.40%)
No-report	30 (13.70%)
Causes of pain	
Migraine	65 (29.70%)
Injuries and accidents	12 (5.50%)
Unknown cause	91 (41.50%)
Other	51 (23.30)
Pain location	
Headache	83 (37.90%)
Spinal column/ Musculoskeletal pain	41 (18.70%)
Chest pain	39 (17.80%)
Stomach pain	29 (13.20%)
Other	27 (12.40%)

Before conducting path analysis, the data were assessed for normality, outlier values, and multicollinearity. No evidence of substantial deviations from normality and outliers was observed. Besides, the Pearson correlation test results indicated that the correlations coefficients among variables were lower than 0.90, and multicollinearity was not present (Table 3).

**Table 3:** Bivariate correlations between study variables

	(1)	(2)	(3)	(4)	(5)	(6)	(7)	(8)	(9)
1. Chronic pain	1	0.05^ ns^	0.11**	– 0.03^ ns^	0.18**	0.11**	0.10**	0.12**	0.11**
2. Permissive	–	1	0.41**	– 0.40**	0.29**	0.37**	0.34**	0.35**	0.42**
3. Authoritarian	–	–	1	– 0.02^ ns^	0.44**	0.40**	0.37**	0.38**	0.45**
4. Authoritative	–	–	–	1	0.02^ ns^	– 0.08**	– 0.17**	– 0.19**	– 0.15**
5. Disconnection/Rejection	–	–	–	–	1	0.67**	0.56**	0.54**	0.55**
6. Impaired autonomy/performance	–	–	–	–	–	1	0.63**	0.51**	0.59**
7. Other-Directedness	–	–	–	–	–	–	1	0.59**	0.54**
8. Over vigilance/Inhibition	–	–	–	–	–	–	–	1	0.65**
9. Impaired limits	–	–	–	–	–	–	–	–	1

*ns* non-significant

^**^Correlation is significant at the 0.01 level

## Path analysis results

### Path model 1

**Fig. 1:**
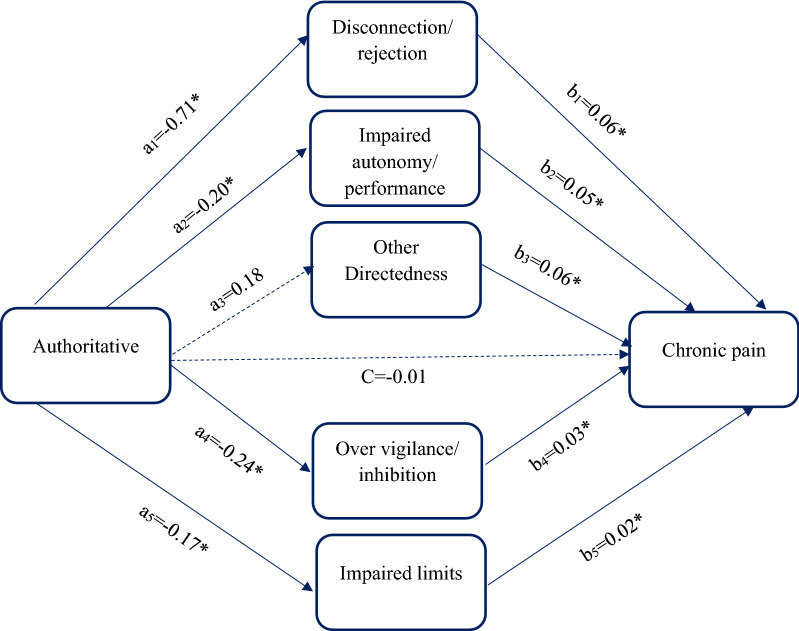
The chart and path coefficients of the mediators in the relationship between authoritative parenting style and chronic pain. Non-significant coefficients are shown with dotted lines; * zero not include in 95% confidence interval. Total effect= -0.01, 95% CI= (-0.25, 0.11)

Path model 1 was performed to evaluate the relationship between authoritative parenting style and chronic pain mediated by disconnection/ rejection, impaired autonomy/ performance, other directedness, over vigilance/ inhibition, and impaired limits (Table 4, Fig. 1). Direct path from authoritative style to chronic pain (β = − 0.010) was not significant (c path). The authoritative style was significantly associated with disconnection/ rejection (β = − 0.71), impaired autonomy/ performance (β = − 0.20), over vigilance/ inhibition (β = − 0.24), and impaired limits (β = − 0.17), whereas the direct path from authoritative style to other directedness (β = 0.18) was not significant (a paths).The direct effects of disconnection/ rejection (β = 0.06), impaired autonomy/ performance (β = 0.05), other directedness (β = 0.06), over vigilance/ inhibition (β = 0.03), and impaired limits (β = 0.02) on chronic pain were significant (b paths). The results in the first model revealed that the indirect path from authoritative style to chronic pain was significant through disconnection/ rejection (β = -0.043), impaired autonomy/ performance (β = − 0.01), over vigilance/ inhibition (β = -0.007), and impaired limits (β = − 0.004). The indirect path from authoritative style to pain via other directedness (β = 0.06) was not significant. The total effect of authoritative style on chronic pain (β = − 0.01) was not significant (Fig. 1, Table 4). The first path model was a good fit (RMSEA = 0.026, CFI = 0.999, TLI = 0.981, χ^2^/df = 48.90, SRMR = 0.10).

**Table 4:** Results for authoritative parenting style as the predictor, the parallel mediators (maladaptive schema), and binary outcome (chronic pain)

Path	Estimate	SD	95% CI	
			Lower	Upper
Direct effect				
a_1_	− 0.71*	0.17	− 1.05	− 0.37
a_2_	− 0.20*	0.06	− 0.33	− 0.06
a_3_	0.18	0.99	− 0.95	2.92
a_4_	− 0.24*	0.03	− 0.30	− 0.18
a_5_	− 0.17*	0.03	− 0.23	− 0.12
b_1_	0.06*	0.01	0.05	0.07
b_2_	0.05*	0.004	0.04	0.06
b_3_	0.06*	0.02	0.03	0.09
b_4_	0.03*	0.01	0.02	0.04
b_5_	0.02*	0.004	0.01	0.03
c	− 0.01	0.01	− 0.01	0.02
Indirect effect				
a_1_b_1_	− 0.043*	0.01	− 0.07	− 0.02
a_2_b_2_	− 0.01*	0.03	− 0.02	− 0.003
a_3_b_3_	0.06	0.06	− 0.18	0.06
a_4_b_4_	− 0.007*	0.002	− 0.01	− 0.008
a_5_b_5_	− 0.004*	0.001	− 0.006	− 0.002
Total effect	− 0.01	0.07	− 0.25	0.11

SD, standard deviation; 95% CI, 95% confidence intervals for estimated path coefficients

*zero not include in 95% confidence interval

a_1_ represents the direct path from authoritative style to disconnection/ rejection; a_2_ represents the direct path from authoritative style to impaired autonomy/ performance; a_3_ represents the direct path from authoritative style to other directedness; a_4_ represents the direct path from authoritative style to over vigilance/ inhibition; a_5_ represents the direct path from authoritative style to impaired limits; b_1_ represents the direct path from disconnection/ rejection to chronic pain; b_2_ represents the direct path from impaired autonomy/ performance to chronic pain; b_3_ represents the direct path from other directedness to chronic pain; b_4_ represents the direct path from over vigilance/ inhibition to chronic pain; b_5_ represents the direct path from impaired limits to chronic pain; c represents the direct path from authoritative style to chronic pain; a_1_b_1_ represents the indirect path from authoritative style to chronic pain through disconnection/ rejection; a_2_b_2_ represents the indirect path from authoritative style to chronic pain through impaired autonomy/ performance; a_3_b_3_ represents the indirect path from authoritative style to chronic pain through other directedness; a_4_b_4_ represents the indirect path from authoritative style to chronic pain through over vigilance/ inhibition; a_5_b_5_ represents the indirect path from authoritative style to chronic pain through impaired limits

### Path model 2

**Fig. 2:**
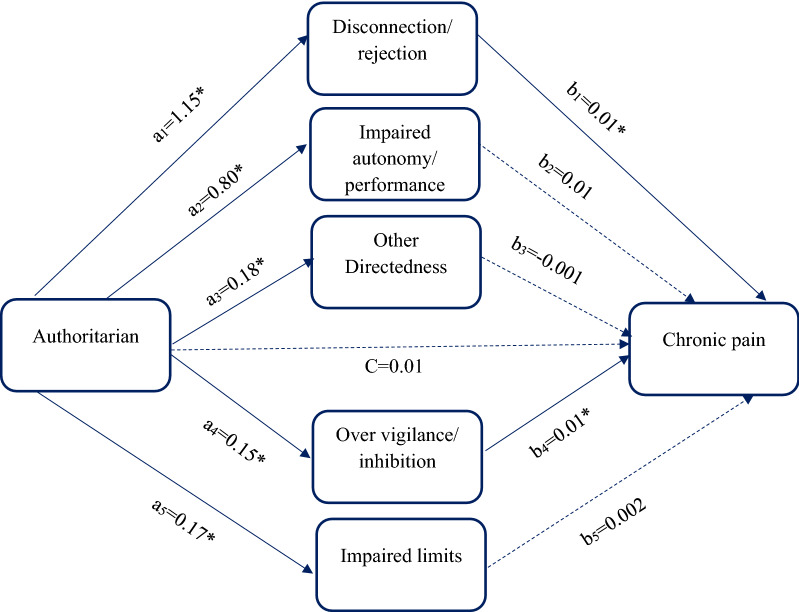
The chart and path coefficients of the mediators in the relationship between authoritarian parenting style and chronic pain. Non-significant coefficients are shown with dotted lines; * zero not include in 95% confidence interval. Total effect= 0.02, 95% CI= (0.01, 0.03)

Path model 2 was conducted to evaluate whether the relationship between authoritarian parenting style and chronic pain was mediated by disconnection/ rejection, impaired autonomy/ performance, other directedness, over vigilance/ inhibition, and impaired limits (Table 5, Fig. 2). The direct effect of authoritarian style on chronic pain (β = 0.01) was not significant (c path). The authoritarian style was significantly associated with disconnection/ rejection (β = 1.15), impaired autonomy/ performance (β = 0.80), other directedness (β = 0.18), over vigilance/ inhibition (β = 0.15), and impaired limits (β = 0.17) (a paths). The direct effects of disconnection/ rejection (β = 0.01) and over vigilance/ inhibition (β = 0.01) on chronic pain were significant, whereas impaired autonomy/ performance (β = 0.01), other directedness (β = − 0.001), and impaired limits (β = 0.002) were not significantly associated with chronic pain (b paths). The results revealed that the indirect path from authoritarian style to chronic pain through disconnection/ rejection (β = 0.01) and over vigilance/ inhibition (β = 0.002) was significant. The indirect path from authoritarian style to chronic pain via impaired autonomy/ performance (β = 0.001), other directedness (β = − 0.0001), and impaired limits (β = 0.0003) was not significant. The total effect of authoritarian style on chronic pain (β = 0.020) was significant (Fig. 2, Table 5). The second path model fitted well (Model fit indices: RMSEA = 0.050, CFI = 0.990, TLI = 0.922, χ2/df = 48.10, SRMR = 0.02).

**Table 5:** Results for authoritarian parenting style as the predictor, the parallel mediators (maladaptive schema), and binary outcome (chronic pain)

Path	Estimate	SD	95% CI	
			Lower	Upper
Direct effect				
a_1_	1.15*	0.06	1.04	1.27
a_2_	0.80*	0.05	0.69	0.90
a_3_	0.18*	0.03	0.13	0.23
a_4_	0.15*	0.03	0.09	0.20
a_5_	0.17*	0.03	0.11	0.22
b_1_	0.01*	0.003	0.01	0.02
b_2_	0.01	0.003	− 0.02	0.01
b_3_	− 0.001	0.01	− 0.01	0.01
b_4_	0.01*	0.01	0.004	0.02
b_5_	0.002	0.01	− 0.01	0.01
c	0.01	0.01	− 0.02	0.003
Indirect effect				
a_1_b_1_	0.01*	0.003	0.01	0.02
a_2_b_2_	0.001	0.003	− 0.001	0.01
a_3_b_3_	− 0.0001	0.001	− 0.002	0.002
a_4_b_4_	0.002*	0.001	0.001	0.02
a_5_b_5_	0.0003	0.001	− 0.002	0.001
Total effect	0.02*	0.005	0.01	0.03

SD, standard deviation; 95% CI, 95% confidence intervals for estimated path coefficients

*zero not include in 95% confidence interval

a_1_ represents the direct path from authoritarian style to disconnection/ rejection; a_2_ represents the direct path from authoritarian style to impaired autonomy/ performance; a_3_ represents the direct path from authoritarian style to other directedness; a_4_ represents the direct path from authoritarian style to over vigilance/ inhibition; a_5_ represents the direct path from authoritarian style to impaired limits; b_1_ represents the direct path from disconnection/ rejection to chronic pain; b_2_ represents the direct path from impaired autonomy/ performance to chronic pain;b_3_ represents the direct path from other directedness to chronic pain; b_4_ represents the direct path from over vigilance/ inhibition to chronic pain; b_5_ represents the direct path from impaired limits to chronic pain; c represents the direct path from authoritarian style to chronic pain; a_1_b_1_ represents the indirect path from authoritarian style to chronic pain through disconnection/ rejection; a_2_b_2_ represents the indirect path from authoritarian style to chronic pain through impaired autonomy/ performance; a_3_b_3_ represents the indirect path from authoritarian style to chronic pain through other directedness; a_4_b_4_ represents the indirect path from authoritarian style to chronic pain through over vigilance/ inhibition; a_5_b_5_ represents the indirect path from authoritarian style to chronic pain through impaired limits.

### Path model 3

**Fig. 3:**
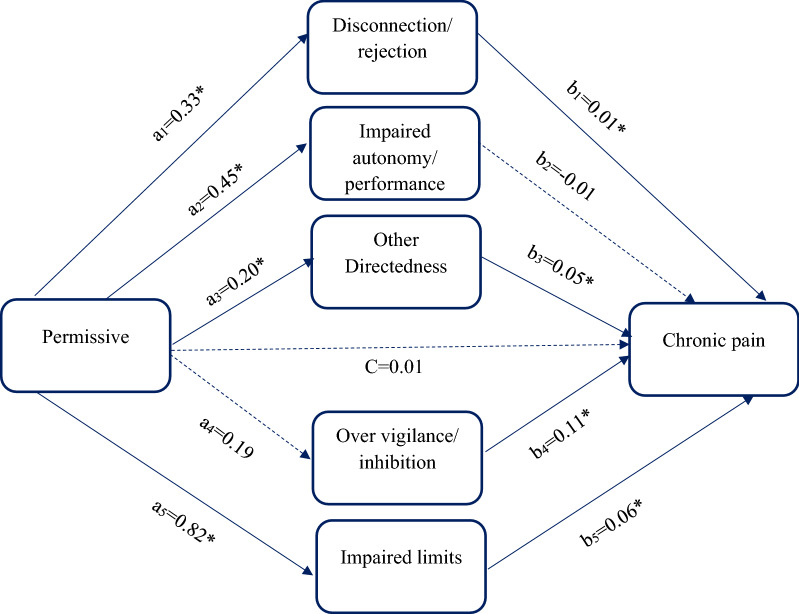
The chart and path coefficients of the mediators in the relationship between permissive parenting style and chronic pain. Non-significant coefficients are shown with dotted lines; * zero not include in 95% confidence interval. Total effect= 0.09, 95% CI= (-0.04, 0.12)

The third path model was designed to explore the relationship between permissive style and chronic pain through the mediators, including disconnection/ rejection, impaired autonomy/ performance, other directedness, over vigilance/ inhibition, and impaired limits (Table 6, Fig. 3). Direct path from permissive style to chronic pain (β = 0.01) was not significant (c path). The direct path was significant from permissive style to disconnection/ rejection (β = 0.33), impaired autonomy/ performance (β = 0.45), other directedness (β = 0.20), and impaired limits (β = 0.82), whereas to over vigilance/ inhibition (β = 0.19) was not significant (a paths). The direct paths were significant from disconnection/ rejection (β = 0.01), other directedness (β = 0.05), over vigilance/ inhibition (β = 0.11), and impaired limits (β = 0.06) to the chronic pain, whereas impaired autonomy/ performance (β = -0.01) was not significantly associated with chronic pain (b paths). The results of path model 3 demonstrated that the indirect paths from permissive style to chronic pain through disconnection/ rejection (β = 0.004), other directedness (β = 0.01), and impaired limits (β = 0.05) were significant. The indirect paths from permissive style to chronic pain through impaired autonomy/ performance (β =− 0.005) and over vigilance/ inhibition (β = 0.02) were not significant. The total effect of permissive style on chronic pain (β = 0.09) was not significant (Fig. 3, Table 6). The path model 3 was a good fit (RMSEA = 0.049, CFI = 0.991, TLI = 0.936, χ^2^/df = 50.53, SRMR = 0.11).

**Table 6:** Results for permissive parenting style as the predictor, the parallel mediators (maladaptive schema), and binary outcome (chronic pain)

Path	estimate	SD	95% CI	
			Lower	Upper
Direct effect				
a_1_	0.33*	0.10	0.14	0.53
a_2_	0.45*	0.14	0.19	0.72
a_3_	0.20*	0.04	0.12	0.27
a_4_	0.19	0.28	− 0.37	0.74
a_5_	0.82*	0.05	0.72	0.91
b_1_	0.01*	0.002	0.01	0.02
b_2_	− 0.01	0.003	− 0.001	0.02
b_3_	0.05*	0.004	0.04	0.05
b_4_	0.11*	0.01	0.10	0.12
b_5_	0.06*	0.003	0.05	0.07
c	0.01	0.01	− 0.03	0.01
Indirect effect				
_1_b_1_	0.004*	0.001	0.001	0.01
a_2_b_2_	− 0.005	0.002	− 0.01	0.001
a_3_b_3_	0.01*	0.002	0.005	0.015
a_4_b_4_	0.02	0.03	− 0.04	0.08
a_5_b_5_	0.05*	0.004	0.04	0.06
Total effect	0.09	0.04	− 0.04	0.12

SD, standard deviation; 95% CI, 95% confidence intervals for estimated path coefficients

*zero not include in 95% confidence interval

a_1_ represents the direct path from permissive style to disconnection and rejection; ${a}_{2}$ represents the direct path from permissive style to impaired autonomy and performance; ${a}_{3}$ represents the direct path from permissive style to other directedness; a_4_ represents the direct path from permissive style to over vigilance and inhibition; a_5_ represents the direct path from permissive style to impaired limits; ${b}_{1}$ represents the direct path from disconnection and rejection to chronic pain; ${b}_{2}$ represents the direct path from impaired autonomy and performance to chronic pain; ${b}_{3}$ represents the direct path from other directedness to chronic pain; b_4_ represents the direct path from over vigilance and inhibition to chronic pain; b_5_ represents the direct path from impaired limits to chronic pain; c represents the direct path from permissive style to chronic pain; ${a}_{1}{b}_{1}$ represents the indirect path from permissive style to chronic pain through disconnection and rejection; ${a}_{2}{b}_{2}$ represents the indirect path from permissive style to chronic pain through impaired autonomy and performance; ${a}_{3}{b}_{3}$ represents the indirect path from permissive style to chronic pain through other directedness; a_4_b_4_ represents the indirect path from permissive style to chronic pain through over vigilance and inhibition; a_5_b_5_ represents the indirect path from permissive style to chronic pain through impaired limits.

## Discussion

Chronic pain seriously affects the daily lives of adolescents and imposes a heavy financial burden on the society [65, 66]. Most adolescents who suffer from a chronic pain face many problems such as a low level of education and a weak professional performance in their adulthood [67]. Therefore, it seems that determining the elements that contribute to the development or exacerbation of chronic pain in adolescents might lead to the mitigation of the consequences of this prevalent disease. The current study aimed to explore whether parenting styles are associated with chronic pain through maladaptive schemas.

According to the results of this study, disconnection/ rejection, impaired autonomy/ performance, over-vigilance/ inhibition, and impaired limits schemas significantly mediated the protective effects of the authoritative parenting style on chronic pain. It was also found that the mediating effects of disconnection/ rejection and over-vigilance/ inhibition existed in the relationship between the authoritarian style and chronic pain. In contrast to our hypothesis, the permissive style may also affect chronic pain through disconnection/ rejection, other-directedness, and impaired limits schemas.

The findings of the current study are consistent with Palermo & Chambers’ model which states that adolescents' emotional functioning plays a mediating role in the relationship between family functioning and pain [31]. According to Young schema therapy model, unpleasant childhood experiences (mainly caused as the result of interacting with parents) could lead to the development of long-standing emotional disorders in individuals through creating some maladaptive schemas [34]. Our findings may offer one piece of the missing evidence through providing some evidence to support the effects of family functioning and unpleasant childhood experiences regarding pediatric chronic pain. However, further studies are needed to extend the existing knowledge about the possible dependence and/or the potential relationship between the parenting style and chronic pain.

### Parenting styles in relation to maladaptive schemas

The way parents interact properly with their children can have a positive effect on their children's mental health by reducing the development of maladaptive schemas [34]. In the authoritative parenting style, by establishing a close relationship with children, adequate supportive and loving behaviors, proper control and setting specific rules, parents would raise children who have self-confidence, are securely attached, and interact with peers using competent social skills. Accordingly, as can be seen in our results, authoritative parenting style may play a protective role against the development of the disconnection/ rejection domain. Authoritative parents have less sudden impulses of anger and aggression. Thus, as our results show, their adolescents are protected against over vigilance/inhibition domain. Such parents are characterized as providing clear and firm direction for their children, thus, protecting their children from impaired limits domain. In addition, because authoritative parents encourage independence in their children, they learn that they are able to achieve their goals independently [68]. Therefore, as can be seen in the present findings, authoritative parenting style may play a protective role against the development of impaired autonomy/ performance domain. In line with the current results, some of the previous studies have also shown a relationship between the authoritative parenting style and a reduced likelihood of developing the maladaptive schemas of disconnection and rejection [69], impaired autonomy/ performance [70], and over -vigilance/ inhibition [36].

Conversely, the behavioral pattern of the authoritarian parents includes too much control along with too little love. Therefore, the children cannot establish a secure and satisfying emotional attachment and relationship with others. Thus, as our results indicate, such a pattern of behavior can predisposes children to the development of the disconnection/ rejection domain [68]. Authoritarian parents usually set severe and strict rules for their children and expect their children to obey these rules without any questions. The rules are usually not explained and it is not possible for the children to negotiate these rules. Any committed mistake by the children is not accepted by the parents and if it happens, the children will be punished. High expectations, low flexibility, and sudden outbursts of anger are also common in such families. As a result, these children learn to be perfect and constantly try to suppress their emotions. Such a pattern of behavior can predispose children to the development of the over-vigilance/inhibition domain. There are many studies, which are in line with the results of the present study, showing the association of affectionless control parenting with the higher levels of maladaptive schemas of disconnection and rejection, and over -vigilance/ inhibition [36, 38, 39, 69–71].

Permissive parenting style, such as the authoritarian parenting style, has a negative impact on children's mental health. However, these impacts can also be influenced to some extent by cultural factors [72]. The behavioral pattern of the permissive parents is associated with high love and low control. Such parents usually impose a few or no rules in the family. It is natural that there will be no order and discipline in these families and the children will have lots of freedom to make decisions in various matters of life. In addition to excessive freedom, the children will develop negative habits and unhealthy lifestyles because parents do not provide the necessary guidance to them. Since the children of such parents have not learned how to cope with their problems, they generally become irresponsible, careless, expectant, selfish and have a sense of superiority over others. Deficiency in internal limits, responsibility to others, or long-term goal orientation leads to difficulty respecting the rights of others and cooperating with others. Thus, as our results indicate, permissive parenting can predispose children to the development of the impaired limits domain. Of course, it is also possible for such children to become confused, especially in identity, due to the negligence and permissiveness of the parents and not be able to correctly identify their needs and feelings or even not to decide for themselves independently. As a result, they obey and accept the behaviors and orders of other people, respectively [73]. Accordingly, as can be seen in our results, the likelihood of other-directedness is high among the children of such families. Our findings have also shown a relationship between the permissive parenting style and an increased likelihood of developing the maladaptive schemas of disconnection and rejection. Some research has proposed that when parents are too permissive and don’t take charge of their children, the children might take on the role of a parent [74]. This change of roles may create conflict between children and parents, which, in turn, could lead to child-to-parent aggression [75]. According to the schema therapy model, exposure to aggression and violence can lead to the development of disconnection and rejection schemas [75]. Consistent with these results, Adibsereshki et al. showed a significant relationship between the permissive parenting style and the maladaptive schemas of disconnection and rejection, and impaired limits [70]. However, unfortunately, our measure of parenting style did not distinguish between indulgent and neglecting parents and the results need to be replicated using instruments assigning the parents to four categories (i.e. authoritative, authoritarian, permissive and neglectful) based on their parenting style.

### The relationship between maladaptive schemas and chronic pain

Cognitive schemas may affect physical health, particularly chronic pain, in adolescents due to the development of negative and maladaptive emotions [76]. However, few studies have investigated the mechanism of the effects of cognitive schemas on chronic pain. It seems that schemas may affect the development/extension of pain in individuals in a variety of ways. In their study, Vakili et al. reported that in patients with chronic pain, maladaptive schemas generally affect chronic pain through depression [40]. Davoodi et al. also showed the relationship of all 5 areas of early maladaptive schemas with depression [77]. It is well known that depression can affect the development/ severity of chronic pain [78]. In addition, Brown posited that cognitive schemas can affect an individual’s perception and attention which may result in the misinterpretation of the sensations and bodily signals [79]. Consistently, Riebel et al. showed that patients with somatoform disorders indicated self-concepts of being weak and inability to tolerate stress [80]. Therefore, it seems that cognitive schemas can play an important role in causing or perpetuating pain by misinterpreting the bodily sensations as well as catastrophizing the physical symptoms.

Furthermore, according to cognitive models of psychosis, early maladaptive schemas can lead to psychological vulnerability such as anxiety, depression, obsessive compulsive disorder (OCD) and internet addiction in individuals [77, 81, 82] which are in turn considered the risk factors in developing chronic pain [83–85]. Cognitive schemas can also affect a person's ability to express his/her emotions effectively and to communicate efficiently with others. Moreover, cognitive schemas can affect self-esteem and psychological distress. On the other hand, it has been shown that emotional intelligence, self-esteem and psychological distress are important predictors of chronic pain in adolescents [10]. Overall, the present findings indicate that raising children with authoritative, authoritarian and permissive parenting styles may affect the various areas of early maladaptive schemas and subsequently the adolescents' pain experience.s

The findings of the current study extend the previous researches by examining some novel models regarding the relationship between parenting styles and chronic pain in adolescents.

The findings of the current study have some useful applications for both families and teachers. It seems that a close and loving relationship along with appropriate control of children and emotional support for them can be considered as one of the most important ways to reduce the likelihood of the development or severity of chronic pain in adolescents. Parents’ appropriate behavioral patterns can play important roles in improving adolescents' physical health by creating a sense of worth, strengthening independence, and reducing unrealistic expectations of the adolescents. Parents and teachers need to be able to understand the value of supporting adolescents. In this regard, teaching the correct methods of the parenting styles as well as appropriate ways for interacting with adolescents are very important. In addition, the findings of the current study can be useful in the processes regarding the family and group therapies for families having adolescents who suffer from chronic pain.

It is necessary to mention some limitations of the current study. Chronic pain was assessed based on a self-report questionnaire and not a clinical examination. Compared to the clinical examination, the accuracy of measured variables based on a self-report may be influenced to some extent by the response bias [86]. McCaffrey, however, stated that pain is what the sufferer says and lasts as long as he/she feels it [87]. In addition, taking the cross-sectional nature of the design into consideration, findings of the current study do not clarify the causal relationships. More prospective longitudinal studies are needed to elucidate the contribution of adolescents’ psychological characteristics and the parenting styles to the chronic pain. As the questionnaires were completed online, can’t be sure that adolescents actually completed them and that they were alone to do that, especially given the assessment of parenting styles. In the current study, we used the very common Baumrind approach with only three parenting styles (namely authoritative, authoritarian and permissive), which has some limitations, despite that it is not an incorrect approach. An extension of this research would be to investigate whether negligent parenting style (the fourth parenting style added by Maccoby & Martin) may influence chronic pain in adolescents. In addition, undoubtedly, there are factors other than those evaluated in this study that affect chronic pain in adolescents. Therefore, it is recommended to conduct more studies and to evaluate other psychosocial mediating variables that have not been examined in this study. Furthermore, it is recommended to carry out this study on a variety of other samples with age variations and different cultures in order to test the repeatability of the results.

## Conclusion

Due to the increasing prevalence of chronic pain in adolescents in recent years, it is necessary to identify the factors that will have some kinds of influences on chronic pain. Among the effective factors, one can refer to the behavioral patterns of parents with adolescents. However, most of the previous studies focused on the role of these patterns on the psychological state of adolescents and not on the chronic pain. Therefore, the current study was conducted with the aim of answering the question "Do maladaptive cognitive schemas play a mediating role in the relationship between parenting styles and chronic pain in adolescents?" The findings of this study showed that parenting styles could affect the chronic pain in adolescents through creating or preventing the development of the maladaptive schemas.

It seems that the interventions that target the effective communication between the parents and the adolescents can be considered as an important part in controlling chronic pain in adolescents. In addition, the present study would increase our knowledge about the effect of maladaptive cognitive schemas on chronic pain. In regard to, familiarity with the maladaptive schemas, understanding the importance of using the effective parenting styles, acquiring necessary knowledge and skills to support adolescents and to meet their psychological needs are some of the characteristics which should be considered to be very important for parents. In general, based on the findings of the present study, cognitive-behavioral training programs for improving the parenting practices and correcting or reducing the maladaptive schemas for parents and adolescents can be considered very important strategies to reduce the likelihood of causing or exacerbating chronic pain in adolescents.

## Data Availability

The datasets used and/or analyzed during the current study are available from the corresponding author on reasonable request.

## Abbreviations

ICD-11: 11Th revision of the International Classification of Diseases
YSQ-SF: Young’s Schema Questionnaire-Short Form
PAQ: Parental Authority Questionnaire
CFI: Comparative Fit Index
RMSEA: Root Mean Square Error of Approximation
TLI: Tucker–Lewis Index
